# Erythrocyte glutathione levels as long-term predictor of transition to psychosis

**DOI:** 10.1038/tp.2017.30

**Published:** 2017-03-21

**Authors:** S Lavoie, M Berger, M Schlögelhofer, M R Schäfer, S Rice, S-W Kim, J Hesse, P D McGorry, S Smesny, G P Amminger

**Affiliations:** 1 Orygen, The National Centre of Excellence in Youth Mental Health, Parkville, VIC, Australia; 2 Centre for Youth Mental Health, The University of Melbourne, Parkville, VIC, Australia; 3 Laboratory of Psychiatric Neuroscience, Australian Institute of Tropical Health and Medicine (AITHM), Douglas, QLD, Australia; 4 Department of Child and Adolescent Psychiatry, Medical University of Vienna, Vienna, Austria; 5 Department of Psychiatry, Chonnam National University Medical School, Gwangju, Republic of Korea; 6 Department of Dermatology, University Hospital Jena, Jena, Germany; 7 Department of Psychiatry, University Hospital Jena, Jena, Germany

## Abstract

A high proportion of individuals deemed at elevated risk for psychosis will actually never progress to develop the illness. Pharmaceutical intervention may not be necessary in these cases, and may in fact be damaging depending on the invasiveness of the treatment strategy. This highlights the need for biomarkers that are better able to reliably differentiate between at-risk individuals who will subsequently transition to psychosis and those who will not. Low glutathione (GSH) levels have been observed in schizophrenia and in patients with first-episode psychosis. The aim of this study was to determine the predictive value of erythrocyte GSH levels on the transition to psychosis in individuals at risk of developing the illness. Erythrocyte GSH levels were measured in 36 at-risk individuals, 15 of whom had transitioned to psychosis at the 7-year follow-up. Univariate Cox regression analysis showed that transition to psychosis at the 7-year time point was significantly associated with low GSH levels at baseline. The area under the receiving operating characteristic curve was 0.819, indicating that GSH can be considered a good predictor of outcome. Although these results need to be replicated, adding the criterion ‘low erythrocyte GSH' to the set of criteria used to identify individuals at risk of psychosis may be indicated.

## Introduction

Young people who experience attenuated psychotic symptoms, brief limited intermittent psychotic symptoms or possess a key risk factor (for example, schizotypal personality disorder, first-degree relative with psychosis), along with a drop in psychosocial functioning, are at increased risk of developing a psychotic disorder.^1, 2^ However, although this ultra-high risk (UHR) population shows a higher-than-average risk of developing acute psychotic disorders, long-term follow-up (10 years) indicates that a high proportion (approximately 65%) of these individuals does not transition to psychosis.^3, 4^ Pharmaceutical intervention may not be necessary in some cases, and may in fact be damaging depending on the invasiveness of the treatment strategy.^5^ This highlights the urgent need for biomarkers that are helpful to improve the differentiation between UHR individuals who subsequently will transition to psychosis (UHR-T), and those who will not (UHR-NT). The discovery of a reliable biomarker for predicting transition to psychosis in UHR individuals would be instrumental for targeting and improving the effectiveness of early detection and intervention.

Glutathione (GSH) is a major cellular redox regulator and antioxidant, protecting cells from damage induced by reactive oxygen species. GSH levels have been shown to be decreased in the brain,^6, 7^ cerebrospinal fluid,^6^ erythrocytes^8, 9^ and plasma^10^ of schizophrenia patients. In patients with first-episode psychosis (FEP), decreased GSH levels were also observed in erythrocytes^11^ and plasma.^12^ Furthermore, in early psychosis, as well as in chronic schizophrenia, the GSH deficit has been associated with a genetic factor, namely a trinucleotide repeat polymorphism in the gene coding for the catalytic subunit of glutamate-cysteine ligase, the rate-limiting enzyme for GSH synthesis.^13, 14^ Presenting with low GSH levels could, therefore, be an indicator of elevated risk for psychotic disorders.

To our knowledge, the predictive value of erythrocyte GSH levels on the transition to psychosis in UHR individuals has never been tested, and therefore, this is the aim of the current study.

## Materials and methods

### Participants

The participants were recruited in the context of a randomized controlled trial with omega-3 polyunsaturated fatty acids (PUFAs) or placebo intervention (for details, see ref. 15, registration number NCT00396643). The 12-week intervention period was followed by assessments at 12.5±0.9 (mean±s.d.) months and 7.2±0.8 years after the start of treatment. The trial was performed at the Medical University of Vienna and approved by the Medical University of Vienna Ethics Committee. Written informed consent was obtained from every participant.

Individuals were eligible for participation if they were aged 13 to 25 years and met criteria for one or more of the three operationally defined and well-validated groups of risk factors for psychosis,^16, 17^ (i) attenuated positive psychotic symptoms, (ii) transient psychosis and (iii) genetic risk along with a 30% decrease in functioning over the past year. Exclusion criteria included a history of a previous psychotic disorder or manic episode, substance-induced psychotic disorder, acute suicidal or aggressive behavior, current DSM-IV diagnosis of substance dependence (except cannabis dependence), neurological disorders and an intelligence quotient (IQ) of less than 70. People with gross structural brain abnormalities observable on their MR scan were excluded. Also excluded were people who had previous treatment with an antipsychotic or mood-stabilizing agent for more than one week and people who had taken omega-3 PUFA supplements within 8 weeks of being included in the trial. Finally, people showing laboratory values more than 10% outside the normal range for transaminases, thyroid hormones, C-reactive protein or bleeding parameters were excluded.

Out of the 256 individuals who were assessed for eligibility, 81 met the inclusion criteria and consented to the study. After randomization, 40 participants received omega-3 PUFAs and 41 participants received placebo. Because the conversion rates differed significantly between the treatment groups in this trial at both the 12-month^15^ and the 7-year^18^ follow-up, only those participants who received placebo were included in the present study in order to eliminate treatment effects.

### Glutathione measurements

The blood samples were collected at baseline and erythrocyte lysates were stored at −20 °C. GSH levels were measured in batches within 6 months of data collection. The total GSH concentrations were determined in fourfold diluted erythrocyte lysates by means of a commercially available test kit (Cayman Chemical, Ann Arbor, MI, USA; Catalog no. 703002) according to the manufacturer's instructions. The results are expressed in μmol l^−1^ (μm) lysate. Note that because the manufacturer cannot guarantee the accuracy of the oxidized GSH content in erythrocytes, these results are not presented here.

GSH measurement is missing in five out of the 41 participants, including one who transitioned to psychosis.

### Psychopathology

At baseline, symptom severity was quantified using the Positive and Negative Syndrome Scale (PANSS; see ref. 19) and the Montgomery–Åsberg Depression Rating Scale (MADRS; see ref. 20). General functioning was assessed using the Global Assessment of Functioning (GAF; see ref. 21).

### Statistical analyses

The comparisons between UHR-NT and UHR-T at 12 months and between UHR-NT and UHR-T at 7 years were performed using the two-sided Student's *t*-tests or chi-square tests, where appropriate. The continuous variables were normally distributed and the variance between the groups was similar.

The efficacy of blood GSH levels to predict transition to psychosis at the 12-month and 7-year follow-up time points was tested using univariate Cox regressions. When Cox regressions showed significant predictive values of GSH, ROC analysis was performed to find out the accuracy.

Pearson's correlations were used to look at the relationship between GSH levels and symptoms severity and functioning.

The investigators were blinded at the time of data collection and GSH analyses, but they were unblinded for statistical analyses.

## Results

The patients' characteristics are summarized in Table 1. Out of the 41 participants who were included in the placebo group, 36 had their GSH levels measured at baseline, and therefore, 21 UHR-NT, 11 UHR-T at 12-month follow-up and 15 UHR-T at 7-year follow-up (including the 11 who had already transitioned at 12 months) were included in the analyses for the current study. There was no difference between gender distribution, age, at risk for psychosis group belonging at intake and medication at baseline between UHR-NT and the UHR-T groups. The PANSS global and total, as well as GAF scores were lower in the UHR-T group compared with UHR-NT. The PANSS negative score was higher in UHR-T only at the 12-month time point, whereas PANSS positive and MADRS scores were statistically the same in all the three groups. The GSH levels were significantly lower in the UHR-T group at 7-year (*n*=15) compared with UHR-NT (*P*<0.001). However, at the 12-month interval, this difference between UHR-NT and UHR-T (*n*=11) only reached trend level (*P*=0.065).

Univariate Cox regression analysis showed that transition to psychosis at the 7-year time point was significantly associated with low GSH levels at baseline (Table 2). The low GSH levels at the 12-month follow-up did not reach significance as a predictor of psychosis (Table 2).

**Figure 1:**
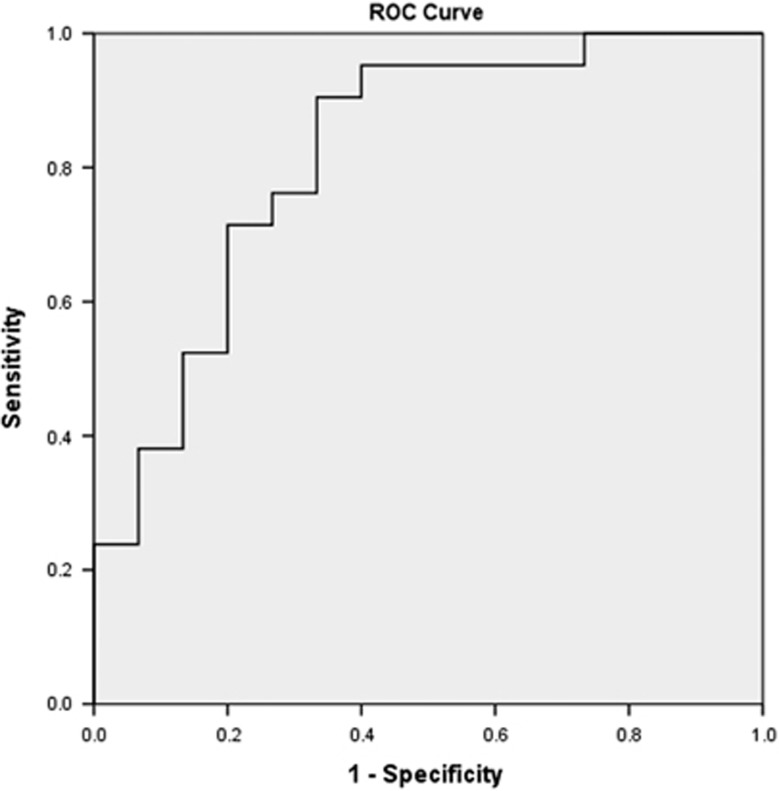
AUROC curve for GSH levels in differentiating between UHR individuals who had transitioned to psychosis and those who did not at the 7-year follow-up time point. AUROC, area under the receiving operating characteristic curve; GSH, glutathione; UHR, ultra-high risk.

**Figure 2:**
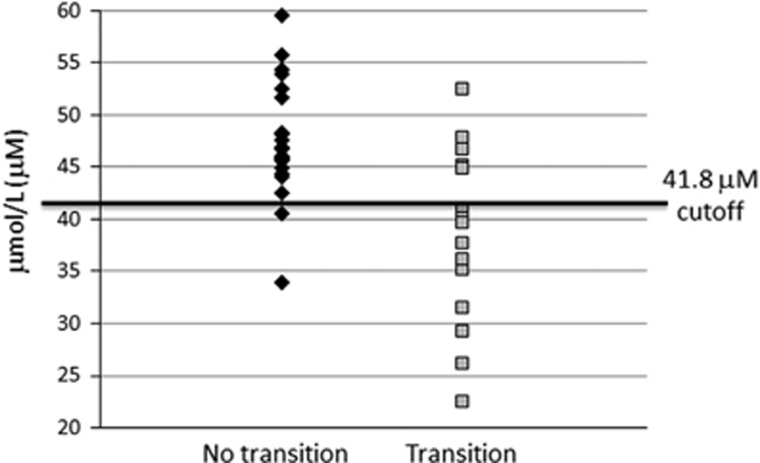
Erythrocyte GSH levels according to transition to psychosis status at the 7-year follow-up time point. With a cut-off of 41.8 μm, 83.8% of the UHR with low GSH levels would have been true positives. GSH, glutathione; UHR, ultra-high risk.

The area under the receiving operating characteristic curve depicted in Figure 1 was 0.819, which indicated that GSH can be considered a good predictor of outcome as measured at the 7-year time point. With a cut-off of 41.8 μm, the sensitivity is 0.905 and specificity is 0.667. Figure 2 shows that the positive predictive value was 83.3%, that is, 10 of the 12 UHR participants classified as ‘low GSH' transitioned to psychosis. The negative predictive value was 79.2%, that is, 19 out of the 24 UHR individuals who showed erythrocyte GSH levels higher than 41.8 μm did not transition to psychosis.

There was no correlation between GSH and any of the symptom scores as assessed with the PANSS, MADRS or GAF ratings at baseline (data not shown). The correlations remained not significant when splitting the group into UHR-NT and UHR-T (data not shown).

## Discussion

Our results show that, in this UHR population, low erythrocyte GSH levels are good predictors of transition to psychosis. In our cohort, adding low erythrocyte GSH levels (<41.8μm) to the UHR criteria would increase the true positive rate from 39 to 83.3%.

Based on the present data, low GSH levels appear to be a good predictor of long-term psychosis outcome, rather than short term. Indeed, at the 12-month time point, the predictive value was only at trend level (present study and ref. 22). Furthermore, the difference in GSH levels between UHR-NT and UHR-T as assessed at the 12-month follow-up was only at trend level. This difference became significant when assessed at the 7-year follow-up. According to our results, reduced GSH levels can be observed as long as 7 years before a FEP, offering the possibility of improved indicated prevention. In UHR individuals who present with low GSH, an agent that enables GSH synthesis, such as *N*-acetylcysteine, might be considered for prevention.^23, 24^

The GSH system represents the most important antioxidant defense of the brain. GSH neutralizes potentially harmful reactive oxygen species produced during normal biochemical processes such as cellular respiration, or following nicotine use and negligent diet, or in the context of mental and somatic disorders. Decreased GSH levels have been observed in the brain of schizophrenia patients^6, 7^ and also in the blood of schizophrenia and FEP patients.^8, 9, 10, 11, 12^ In a balanced cellular system, the homeostasis of GSH is tightly associated with reactive oxygen species levels^25^ that have also been found to be increased in patients with established diagnosis of schizophrenia (for a review, see ref. 26).

Beside increased nicotine use or careless diet, a number of possible causes may explain the decreased GSH levels in UHR patients, and they are all closely related to the pathophysiology of psychosis itself. (i) Following our initial hypothesis, GSH decrease could be explained by genetic defects acting on GSH synthesis as mentioned above.^13, 27^ (ii) Second, dysregulation of the hypothalamic–pituitary–adrenal axis might be linked to impaired functioning of the GSH antioxidant defense system. Indeed, in schizophrenia, chronic hyperactivation of the hypothalamic–pituitary–adrenal axis can be observed (for a review and meta-analyses, see refs 28, 29, 30). Plasma cortisol levels, which reflect the activity of the hypothalamic–pituitary–adrenal axis have also been shown to be increased in UHR Individuals,^31, 32, 33^ especially in UHR-T.^34^ Prolonged increase of cortisol secretion generally reflects a state of chronic stress that, in turn, might lead to increased oxidative stress as reflected by increased reactive oxygen species levels (meta-analysis by Costantini *et al.*^35^). If latent hypercortisolism occurs as early as in the risk phase of psychosis, this could contribute to oxidative stress and successively decreased GSH levels. (iii) Finally, genetic alterations of cellular respiration have been reported in schizophrenia (for a review, see ref. 36) and might therefore be discussed in the context of GSH decrease in UHR individuals.

The pathomechanism of GSH dysfunction in the context of psychosis has not been resolved yet. Compensatory mechanism could involve the upregulation of the synthesis of other antioxidants. However, exploratory analyses on the α-tocopherol (vitamin E) and total tocopherols levels measured in the same cohort did not reveal any difference between UHR participants showing low GSH vs participants with high GSH levels (data not shown). To improve the understanding of mechanisms that might cause oxidative stress and/or decreased GSH in UHR states of psychosis, conjugated follow-up assessments of markers of antioxidant defense, hypothalamic–pituitary–adrenal axis and cellular respiration are granted.

Blood GSH levels measured in FEP patients were shown to be related to the loss of cortical gray matter over a 2-year period.^37^ Furthermore, chronic dysregulation of the antioxidant defense and lipid peroxidation balance was found to be associated with the dysregulation of glutamatergic and dopaminergic systems, which explains most of the psychotic symptoms observed in schizophrenia (for a review, see ref. 38). Following treatment with *N*-acetylcysteine, a precursor of GSH, symptoms were improved in patients with chronic schizophrenia.^39, 40^ Therefore, it was expected in this study to see increased severity of symptoms in people with low GSH, which was not the case. Of those studies that have shown decreased GSH in schizophrenia,^6, 7, 8, 9^ only Raffa *et al.*^10^ observed an inverse correlation between the levels of GSH and the severity of symptoms. Although both studies that showed low GSH in FEP did not observe a relationship between GSH levels and negative symptoms,^11, 12^ Raffa *et al.*^12^ observed a positive correlation between the GSH and positive symptoms. This contradicted their own results obtained in chronic schizophrenia patients.^10^ Therefore, although GSH is known to fulfill important biochemical functions in the brain, its implication in the psychopathology of schizophrenia is unclear and warrants further investigation.

Treatment with PUFAs, ethyl-eicosapentaenoic acid more specifically, was shown to increase brain GSH levels using magnetic resonance spectrometry methods.^41^ Surprisingly, in the same cohort as the present study, GSH levels were not increased following treatment with omega-3 PUFAs (PUFAs;^42^). In both studies, the ethyl-eicosapentaenoic acid dosage was similar and the duration of treatment was 12 weeks, but GSH levels were measured in the brain in the first study and in the blood in the latter study. GSH has poor blood–brain barrier permeability and transport properties, such that blood levels may not reflect cortical levels. It is possible that erythrocyte GSH levels reflect a more chronic state, while the brain GSH levels are more closely monitored and reflect acute treatment response.

Our sample included 41 young individuals deemed at clinical risk for psychosis based on the presence of attenuated psychotic symptoms (95%) or on the occurrence of Brief Limited Intermittent Psychotic Symptoms (BLIPS) episodes (5%). A total of 39% of these individuals had transitioned to psychosis at the 7-year follow-up. If GSH levels lower than 41.8 μm were added to the UHR criteria at the time of recruitment into the study, based on the present results, 16 individuals would have been selected, and 69% of them would have been true positives. In clinical practice, this would mean that a simple blood test followed by commercially available GSH assays could improve the current UHR criteria.

However, four of the 24 people with high GSH levels (16.7%) would have been false negatives. It is difficult to tell whether this rate can be considered high or low in such a limited sample, but of course the optimal biomarker should yield a false negative rate of 0%. Therefore, more research is necessary to identify the most reliable set of predictive biomarkers that amends the established UHR criteria.

Normal GSH range in the blood have mostly been established from plasma samples. However, as demonstrated by Smesny *et al.*,^42^ GSH levels are much higher in erythrocytes than in the plasma and this is why erythrocytes levels were used in this study. The 41.8 μm cut-off is based on the current sample and more studies will be necessary to determine the ideal cut-off, or perhaps preferably a normal range, to be used in clinical settings. Levels may widely vary depending on the methods used for sample collection, processing, storage and quantification. Therefore, the use of a common methodology will be necessary for the use of such a cut-off, or normal range, as a criterion for the at-risk mental state.

According to the literature, it is not clear whether the peripheral GSH levels directly reflects GSH status in the central nervous system. Low GSH levels in the prefrontal cortex, as measured with magnetic resonance spectroscopy have been associated with a dysregulation of GSH homeostasis under oxidative conditions in FEP.^14^ The GSH homeostasis can be determined by measuring the GSH/oxidized GSH ratio. However, because of the technical difficulties linked to the measurement of the unstable oxidized form of GSH, Xin *et al.*^14^ suggest to assess enzymatic activities of GSH peroxidase (GPx) and GSH reductase (GR), and their ratio, in blood cells instead of the GSH/oxidized GSH ratio. The GPx/GR ratio may be a better marker than GSH levels themselves and more research will be needed to verify this.

Our results show low erythrocyte GSH levels to be a good predictor of transition to psychosis. An important strength of this study is that long-term follow-up data have been collected in the sample. However, the study is limited by its small sample size. Although the present results need to be replicated, it appears that adding the criterion of ‘low erythrocyte GSH' to the set of criteria used to identify individuals at risk of psychosis may be indicated. The findings also provide a rationale for intervention trials testing the efficacy of *N*-acetylcysteine to reduce the transition to psychosis rate in UHR patients.

## Figures and Tables

**Table 1:** Baseline characteristics of participants

*Characteristic*	*UHR-NT* *(*n*=21)*	*UHR-T at 12 m* *(*n*=11)*	*UHR-NT vs UHR-T* *12 m;* P	*UHR-T at 7 y* *(*n*=15)*	*UHR-NT vs UHR-T* *7 y;* P
Gender, females *n* (%)	15 (71.4)	7 (63.6)	0.652^a^	10 (66.7)	0.521^a^
Age (years), mean±s.d.	16.4±1.9	15.9±1.1	0.389^b^	14.9±1.3	0.162^b^
					
*At risk for psychosis group,* n *(%)*			0.537^a^		0.462^a^
Attenuated symptoms	18 (85.7)	10 (90.9)		14 (93.3)	
BLIPS	2 (9.5)	0		0	
Attenuated symptoms+state	1 (4.8)	1 (9.1)		0	
Attenuated symptoms+BLIPS	0	0		1 (6.7)	
					
*Psychiatry medication at baseline,* n *(%)*			0.544^a^		1.000^a^
Antidepressant	14 (66.7)	7 (63.6)		10 (66.7)	
Benzodiazepine or sedative	7 (33.3)	4 (36.3)		5 (33.3)	
					
Blood GSH levels (μm), mean±s.d.	45.4±7.7	39.9±8.	0.065^b^	33.4±7.4	**<0.001**^b^
PANSS positive, mean±s.d.	13.7±3.7	15.5±2.0	0.091^b^	13.0±2.9	0.326^b^
PANSS negative, mean±s.d.	11.9±5.6	17.2±7.8	**0.030**^b^	12.5±6.6	0.068^b^
PANSS global, mean±s.d.	27.1±5.3	34.1±6.8	**0.003**^b^	29.5±6.2	**0.007**^b^
PANSS total, mean±s.d.	52.7±12.0	66.8±15.3	**0.007**^b^	55.0±12.4	**0.021**^b^
MADRS, mean±s.d.	17.1±9.4	22.4±7.2	0.114^b^	20.8±7.0	0.099^b^
GAF, mean±s.d.	64.3±11.9	50.9±11.1	**0.004**^b^	61.3±14.9	**0.014**^b^

Abbreviations: BLIPS, Brief Limited Intermittent Psychotic Symptom; GAF, Global Assessment of Functioning; GSH, glutathione; MADRS, Montgomery–Åsberg Depression Rating Scale; PANSS, Positive and Negative Syndrome Scale; UHR-NT, ultra-high risk individuals who did not transition to psychosis; UHR-T, ultra-high risk individuals who did transition to psychosis.

a Chi-square test.

b Student's *t*-test.

Bold values indicate significant differences between groups.

**Table 2:** Results of univariate Cox regression with GSH levels as a predictor of transition to psychosis assessed at the 12-month and the 7-year follow-up

	*Exp(B)*	*95% CI for Exp(B)*		P
		*Lower*	*Upper*	
12 months	0.941	0.879	1.006	0.083
7 years	0.915	0.865	0.969	**0.003**

Abbreviations: CI, confidence interval; GSH, glutathione.

Bold values indicate significant differences between groups.
